# *Spodoptera frugiperda* (Lepidoptera: Noctuidae) host-plant variants: two host strains or two distinct species?

**DOI:** 10.1007/s10709-015-9829-2

**Published:** 2015-02-19

**Authors:** Pascaline Dumas, Fabrice Legeai, Claire Lemaitre, Erwan Scaon, Marion Orsucci, Karine Labadie, Sylvie Gimenez, Anne-Laure Clamens, Hélène Henri, Fabrice Vavre, Jean-Marc Aury, Philippe Fournier, Gael J. Kergoat, Emmanuelle d’Alençon

**Affiliations:** 1 UM - UMR 1333 DGIMI, Université Montpellier, Place Eugène Bataillon, 34095 Montpellier, France; 2 INRA - UMR 1333 DGIMI, Université Montpellier, Place Eugène Bataillon, 34095 Montpellier, France; 3 INRA - UMR 1062 CBGP (INRA, IRD, CIRAD), Montpellier SupAgro, 755 Avenue du campus Agropolis, 34988 Montferrier-Sur-Lez, France; 4 INRA - UMR 1349, Environment and Plant Protection, Institute of Genetics, Domaine de la Motte, BP 35327, 35653 Le Rheu Cedex, France; 5 INRIA Centre Rennes, Bretagne Atlantique, GenOuest, Campus de Beaulieu, 35042 Rennes, France; 6 Commissariat a l’Energie Atomique (CEA), Institut de Genomique (IG), Genoscope, 2 rue Gaston Crémieux, BP5706, 91057 Evry, France; 7 Laboratoire de Biométrie et Biologie Evolutive, UMR CNRS 5558, Université de Lyon, Université Lyon1, Villeurbanne, France

**Keywords:** Host-races, Microsatellite marker, Post-zygotic isolation, Segregation distortion, *Spodoptera frugiperda*

## Abstract

The moth *Spodoptera frugiperda* is a well-known pest of crops throughout the Americas, which consists of two strains adapted to different host-plants: the first feeds preferentially on corn, cotton and sorghum whereas the second is more associated with rice and several pasture grasses. Though morphologically indistinguishable, they exhibit differences in their mating behavior, pheromone compositions, and show development variability according to the host-plant. Though the latter suggest that both strains are different species, this issue is still highly controversial because hybrids naturally occur in the wild, not to mention the discrepancies among published results concerning mating success between the two strains. In order to clarify the status of the two host-plant strains of *S. frugiperda*, we analyze features that possibly reflect the level of post-zygotic isolation: (1) first generation (F1) hybrid lethality and sterility; (2) patterns of meiotic segregation of hybrids in reciprocal second generation (F2), as compared to the meiosis of the two parental strains. We found a significant reduction of mating success in F1 in one direction of the cross and a high level of microsatellite markers showing transmission ratio distortion in the F2 progeny. Our results support the existence of post-zygotic reproductive isolation between the two laboratory strains and are in accordance with the marked level of genetic differentiation that was recovered between individuals of the two strains collected from the field. Altogether these results provide additional evidence in favor of a sibling species status for the two strains.

**Electronic supplementary material:**

The online version of this article (doi:10.1007/s10709-015-9829-2) contains supplementary material, which is available to authorized users.

## Introduction

Speciation, the process by which an ancestral lineage splits into two or more reproductively isolated lineages, is a central process in evolution. Within the biological species context (Mayr 1942), a fundamental component of this process is reproductive isolation, which may result from pre- and/or post-zygotic barriers. Pre-zygotic barriers occur before fecundation and usually consist of differences between populations in term of habitat, biology or behavior. Post-zygotic barriers exist between related species when fitness of hybrid genotypes is lower than those of parental genotypes. Three different kinds of post-zygotic isolation between individuals from distinct species have been described (Dobzhansky 1970): (1) F1 hybrid lethality, (2) F1 hybrid sterility, and (3) F2 hybrid degeneracy. Although in the case of F2 hybrid degeneracy, post-zygotic isolation is not complete because of the existence of gene flow, it is nonetheless considered as a standard step towards the evolution of complete sterility between species (Coyne and Orr 1989, 2004). Regarding the inviability or sterility of hybrids, Dobzhansky and Muller proposed that they may result from the accumulation of genes that function normally in a pure-species genome but produce epistatic interactions in hybrids (Dobzhansky 1937; Muller 1942). When hybrids between these lineages are obtained, these negative interactions can cause inviability and/or sterility in particular recombinant genotypes, such as they are removed by natural selection from hybrid populations. This nonrandom elimination of specific allelic combinations leads to segregation distortion (or transmission ratio distortion) i.e. significant deviation of allele or genotype frequencies from simple Mendelian expectations. One corollary of this explanation is that loci causing hybrid incompatibility are expected to be located at or near regions of transmission distortion in hybrid populations. This corollary was formerly demonstrated by analyzing crosses between two plant species in the genus *Solanum* (Moyle and Graham 2006). Even more remarkably, nonrandom elimination of specific allelic combinations has also been used to infer the genetic basis of hybrid incompatibility among species (Li et al. 1997; Harushima et al. 2001; Myburg et al. 2004; Maheshwari and Barbash 2011).

In this study we propose to investigate and clarify the status of a noctuid moth currently considered as a single species: the fall armyworm (FAW), *Spodoptera frugiperda* (J.E. Smith). This moth is a widespread and important agricultural pest in the Western hemisphere (Pogue 2002; Barros et al. 2010) which has been defined so far as one species with two plant-related strains (Pashley 1986; Prowell et al. 2004; Meagher et al. 2004), also referred as host forms (Juárez et al. 2014). One strain was originally identified from populations feeding preferentially on corn, cotton and sorghum (corn strain; ‘C strain’), while the other was identified from populations feeding preferentially on rice and on various pasture grasses (rice strain; ‘R strain’) (Pashley 1986; Pashley et al. 1985; Pashley and Martin 1987). Corn and rice strains are morphologically identical but genetically distinguishable using strain-specific molecular markers (Lu et al. 1994; Lu and Adang 1996; McMichael and Prowell 1999; Levy et al. 2002; Nagoshi and Meagher 2003; Meagher and Gallo-Meagher 2003; Arias et al. 2012). Because of the latter it is possible to highlight the fact that both variants occur in sympatry (Pashley 1986; Pair et al. 1986; Machado et al. 2008). The FAW is also an excellent migrator with two putative migration patterns (Nagoshi et al. 2012a), one going from South America to Texas and the second going from the Caribbean to Florida, as inferred from the fact that individuals from South America (Argentina and Brazil) share comparable haplotype frequencies with those collected in Texas as do Caribbean and Florida populations (Nagoshi et al. 2012b). These results suggest that the two strains may occur in sympatry throughout the Americas and the Caribbean. Moreover, a recent population genetic study on individuals belonging to the two strains in South America has revealed that different populations are more structured with respect to their host-plants rather than to their geographical origin (Juárez et al. 2014). Other molecular evidence comes from the results of molecular-based species delimitation analyses, which consistently split sequenced individuals into two putative species clusters corresponding to the corn and rice strains (Dumas et al. 2015). Other analyses also indicate that the two strains have likely diverged more than 2 Myr ago (Kergoat et al. 2012). Finally, several pre-zygotic and post-zygotic incompatibilities are known for the two strains (for a review, see Groot et al. 2010), some of which are highly controversial: among known pre-zygotic barriers is the difference in host-related performances of larvae from each strain, each strain developing better on its original host-plant (Pashley 1988; Whitford et al. 1988). Interestingly this point was also recently disputed because of the results of some studies that have found that the rice strain larvae developed better on corn and sorghum than corn strain larvae (Meagher et al. 2004; Groot et al. 2010). Other known pre-zygotic barriers consist of behavioural isolation due to mating allochronism between the two strains (Pashley et al. 1992; Schöfl et al. 2011) or to pheromone differences in females (Pashley and Martin 1987). Concerning post-zygotic barriers to gene flow, Pashley and Martin (1987) observed that in mating experiments between the C strain females and R strain males, no spermatophores were transferred while reciprocal crosses (RC) gave viable offsprings (Pashley and Martin 1987). Other studies (Whitford et al. 1988; Quisenberry 1991) did not confirm these results, instead evidencing successful crosses in both directions between the two strains. While performing backcrosses, Pashley and Martin found that the RC hybrid females mated with low success with their brothers but not at all with males of either parental strain (Pashley and Martin 1987). The same results were obtained by Whitford et al. (1988) and were recently confirmed by Groot et al. (2010). In the wild, putative hybrids between the two strains (identified as containing mitochondrial DNA from one strain and nuclear from the other) have been found in proportions amounting up to 16 % (Prowell et al. 2004). In addition, the presence of inherited microorganisms able to manipulate the reproduction of their host (Engelstädter and Hurst 2009 for review) may explain discrepancies in the level of reproductive isolation measured in these studies, but has not been explored yet in the case of *S. frugiperda*. In particular, the most widespread effect of the endosymbiont *Wolbachia* is the induction of Cytoplasmic Incompatibility (CI), which results in post-mating reproductive isolation when infected males are crossed with uninfected females (unidirectional CI) or with females infected by a different strain of *Wolbachia* (bidirectional CI). Endosymbiotic bacteria, by reducing gene flows, may thus have a role in the speciation process (Brucker and Bordenstein 2012).

Recently, Velásquez-Vélez et al. (2011) have found post-zygotic isolation for several life-history traits in both strains. Furthermore, they have identified a decrease in the number of hybrid females and a reduction in hybrid fertility in *S. frugiperda*, consistent with Haldane’s rule (Haldane 1922), which corresponds to the decrease of selective value of the heterogametic sex in hybrid progeny from an interspecies cross. It has often been observed in early mechanisms of the process of speciation (Presgraves 2002). There is evidence of the continuous nature of speciation (Nosil 2012) with numerous studies (Rundle and Nosil 2005; Nosil et al. 2005; Funk et al. 2006; De Queiroz 2007; Nosil et al. 2009; Peccoud et al. 2009) indicating that the divergence during this process varies continuously. For example, the strength of reproductive isolation can vary quantitatively along the “continuum” of speciation and groups differing by discrete levels of differentiation can often be identified between populations and well-defined species, like host-races in the framework of ecological sympatric speciation (Berlocher and Feder 2002; Drès and Mallet 2002; Thomas et al. 2003; Blair et al. 2005; Matsubayashi et al. 2010). Host-races were defined as “[…] genetically differentiated, sympatric populations of parasites that use different hosts and between which there is appreciable gene flow […]” (Berlocher and Feder 2002). This definition seems partially congruent with features exhibited by *S. frugiperda* and led us to question: (1) the ability of the two strains to mate and reproduce, and (2) the genetics of the resulting hybrid progeny.

Using laboratory strains of both variants, we measured the ratio of fertile crosses when females of the corn strain were mated with males of the rice strain, and vice versa and showed a reduction in the rate of fertile crosses in the former case. We present the first genetic analysis of reciprocal crosses between the two FAW strains, by following the segregation pattern of a set of microsatellite markers (Arias et al. 2012) in F2 populations. Only few markers showed a Mendelian inheritance. These data suggest existence of hybrid incompatibility between the two strains, and led us to explore the genetic basis of these incompatibilities. As mentioned previously, since distorted markers are often linked to genes involved in hybrid incompatibility, we took advantage of the on-going sequencing project of the two strains genome that we have launched (The FAW International Consortium, in preparation) to carry out a preliminary analysis of the genomic environment of distorted markers. We present candidate regions involved in hybrid incompatibility and discuss the possibility that the two strains correspond to two sister species.

## Materials and methods

We used two laboratory strains of *S. frugiperda*. Those strains were seeded with 30–50 pupae ten and four years ago for the corn and rice strain, respectively. Since then, they have been reared under laboratory conditions (on Poitout artificial diet (Poitout and Bues 1974), at 24 °C with a 16:8 h light:dark (L:D) photoperiod and 40 % R.H.). The individuals that seeded the corn strain came from French Guadeloupe whereas those that seeded the rice strain came from Florida (USA).

### Measure of inter-strain versus within-strain mating efficiency and of sex-ratio of the progeny

**Fig. 1:**
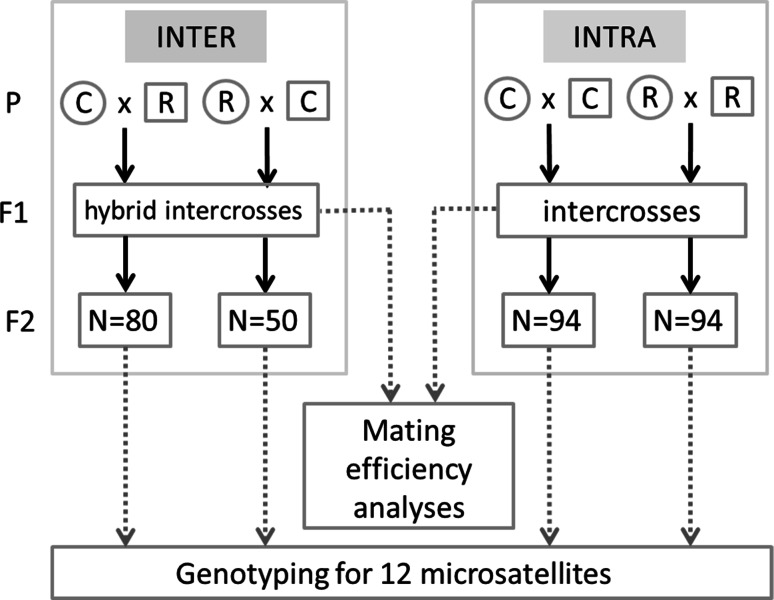
Crossing protocol used to follow microsatellite markers segregation patterns in F2 populations within- and inter-strains. Circles and squares symbolize females and males respectively

Between 27 and 30 isolated couples (using different males and females, to avoid pseudo-replications in the dataset) were constituted for each type of crosses: both C female × R male (one corn female with one rice male; C/R) and R female × C male (one rice female with one corn male; R/C) inter-strain crosses directions and both R female × R male (one rice female with one rice male; R/R) and C female × C male (one corn female with one corn male; C/C) within-strain crosses (Fig. 1). In order to avoid paternity ambiguity, the sex of pupae was anatomically determined before emergence, and pupae of both sexes were reared separately. Virgin females were collected at emergence, and allowed to mate with a single male for five days. The number of couples with hatched larvae was counted and progeny of three couples for each kind of cross were reared in laboratory up to the pupal stage in order to determine sex of pupae. To analyze the ratio of cross giving a viable progeny between all type of cross (i.e. C/C, R/R, C/R, R/C), we used a generalized linear model (GLM) with binomial distribution because the data were binaries (i.e. 0 no progeny, one progeny). The model contained two following factors: the strain female (C or R) and the strain male (C or R), and, we also included the interaction between the two factors (strain female × strain male). Model selection was performed as follow: significance of the different terms was tested starting from the higher-order terms using likelihood-ratio-test (LRT). Non-significant terms (*p* > 0.05) were removed (Crawley 2012). Factor levels of qualitative variables that were not significantly different were grouped (LRT; Crawley, 2012). All computations were performed using the R software version 3.0.3.

### Between strain crosses for microsatellite marker segregation analysis

Two reciprocal F1 intercrosses were obtained: females from C/R crosses x males from the same C/R cross; and the reciprocal R/C × R/C cross (Fig. 1). The number of progeny sampled from each F1 intercross was of 80 and 50 for the C/R and R/C progeny, respectively. In order to avoid paternity ambiguity, sex of pupae was determined before emergence, and pupae of both sexes were reared separately. Virgin females were collected at emergence, and allowed to mate with a single male for five days. Eggs were collected and progeny was reared until the L6 larval instar from which total genomic DNA was extracted by grinding up whole bodies using DNAeasy Blood and Tissue Kit (Qiagen). DNA quality was then assessed using a spectrophotometer (Nanodrop, Wilmington, DE, USA). Finally a total of 130 larvae were typed using microsatellite markers. To do so we used a set of 12 microsatellite markers recently characterized (Arias et al. 2012) to follow microsatellite segregation in the two reciprocal interspecific crosses (starting from either C/R crosses or R/C crosses at G0, at the F2 generation issued from crosses between F1 brothers and sisters). 80 individuals resulting from the C/R reciprocal F1 intercrosses and 50 individuals resulting from the R/C reciprocal were genotyped.

### Within strain crosses for microsatellite marker segregation analysis

Since polymorphism is scarce among individuals of the same strain, the control experiment required a great number of crosses, in order to obtain G0 and F1 parents carrying different alleles of the microsatellites studied. At least 23 pairs of individuals were allowed to mate, and were genotyped at the adult stage after mating, in order to increase the chance of seeing segregation of different alleles of the microsatellite markers in the descendants. Progeny from pairs of the most polymorphic at the different markers analyzed were reared until adulthood corresponding to five pairs (labeled A, B, C, D, E) for the C/C G0 crosses and four pairs (labeled F, G, H, I) for the R/R G0 crosses. At the adult stage, 9, 9, 8, 7, 1 F1 intercrosses issued from pairs A to E, respectively, for the corn strain, and 8, 8, 7, 6 F1 intercrosses issued from pairs F to I, respectively, for the rice strain were performed and genotyped. 94 descendants were genotyped from one of the F1 intercross (rice strain), 63 from a second one.

### Genotyping of F2 laboratory populations (from F1 brotherhood intercross)

Twelve microsatellite markers were typed. The corresponding primer pairs were designed according to (Arias et al. 2012). Their DNA sequences are available in (Arias et al. 2012) except for *Sfrugi11* and *Sfrugi76* which were amplified with the following primer pairs:

- *Sfrugi11* forward specific primer: TGTAAAACGACGGCCAGTGTAAGCAAAAAGCATTTGCCCTA.
- *Sfrugi11* reverse specific primer: TTCCTGACGAACATTCTGGA.
- *Sfrugi76* forward specific primer: TGTAAAACGACGGCCAGTGTATCGTTACCAAGCCGTGC.
- *Sfrugi76* reverse specific primer: ACCCTTATTGGCAATCGAAA.
- Forward fluorescent primer: FAM-TGTAAAACGACGGCCAGT.

Adapting the method of (Schuelke 2000), the 10 µL polymerase chain reactions (PCR) contained 4–10 ng of template DNA diluted in sterile Millipore water, 2 µL of 5 × GoTaq reaction buffer, 1.5 mM MgCl2, 0.2 mM of each dNTP, 0.04 µM of the forward primer (which included a 19 nucleotide tail corresponding to a sequence of the M13 bacteriophage), 0.15 µM of the reverse primer, 0.15 µM of the fluorescence marked M13 primer and 0.1 µL units of recombinant GoTaq-polymerase (5 u/µL, Promega). The PCR protocol included an initial denaturation step at 94 °C for 4 min, followed by 12 cycles involving denaturation at 94 °C for 30 s; annealing at 60 °C for 1 min and extension at 72 °C for 30 s; then 25 cycles of denaturation at 94 °C for 30 s; annealing at 52 °C for 1 min and extension at 72 °C for 30 s; with a final extension step at 72 °C for 10 min. Four 96-well PCR products were simultaneously pooled and diluted to a ratio of 1:75. 2 μL of each PCR were mixed with 0.1 μL of a fluorescent size ladder (GeneScan 500 LIZ) and 15 μL of Hidi Formamide (Applied Biosystems). Electrophoresis and allele detection were carried out on an ABI 3130 automated sequencer. Output was analyzed with Genemapper v.3.7 software (Applied Biosystems, USA). All marker data were verified manually by visual inspection to eliminate errors that may result from the automatic allele assignment procedure following (Piffaretti et al. 2012). To estimate genotype frequencies among members of F2 populations, Chi squared tests were performed using R version 3.0.3.

### Screening for endosymbiotic bacteria

DNA extraction and purification were performed using NucleoSpin Tissue Kit from Macherey–Nagel, following the manufacturer instructions but including a filtration of lysates with NucleoSpin Filters. Elutions were performed using 200 µL of elution buffer provided with the kit. We used different couples of primers (supplementary table S2) to investigate the presence of endosymbiotic bacteria: *Arsenophonus, Hamiltonella, Rickettsia, Wolbachia* and more largely the phylum Bacteroidetes, such as *Cardinium.* For all analyses, a sample infected with the endosymbiont that we seek was used as positive control (see details in supplementary table S2). Note that the primers used for the screening of *Wolbachia*, target at least *Wolbachia* from supergroups A and B, which are the most prevalent clades in insects. Moreover, DNA quality was systemically tested using PCR amplification of the two following genes: Cytochrome c oxidase subunit I (COI) and internal transcribed spacer 2 (ITS2) (supplementary table S2). PCR were performed in a final volume of 25 µL containing 200 µM of dNTP, 200 nM of each primer, 1X Taq buffer and 0.5 U of Taq polymerase (DreamTaq polymerase, Thermo Scientific Fermentas) and 2 µL of the DNA template. PCR were performed under the following conditions: initial denaturation at 94 °C for 2 min, 35 cycles of denaturation (94 °C, 30 s), annealing (temperature depending on primers, 30 s), extension (72 °C, 1 min) and a final extension at 72 °C for 5 min. The PCR products were then visualized using agarose gel electrophoresis. All tests were performed on three individuals of *S. frugiperda* from rice and corn strains.

### Genomic location of distorted markers

Taking advantage of the on-going genome project of *S. frugiperda* (The FAW Consortium, in preparation), we identified the three scaffolds matching with the distorted markers using the Blastn algorithm. *Ab initio* gene annotation was obtained using the fgenesh software (available at http://linux1.softberry.com/) with the parameters set for *Drosophila melanogaster*.

## Results

### Percentage of fertility in F1 crosses between and within corn and rice strains

**Fig. 2:**
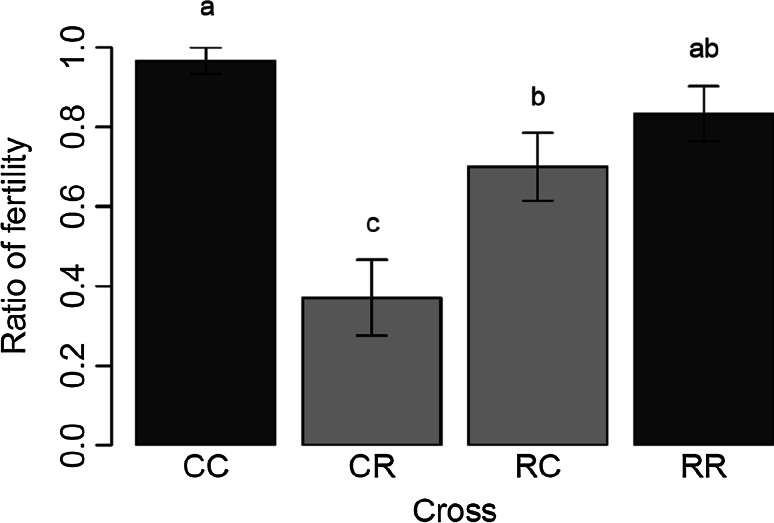
Ratio of fertility in inter-strain crosses (*light grey columns*) in both directions of the cross. C/R (female corn with male rice) and R/C (female rice with male corn) cross direction. The within-strain crosses (*dark grey columns*) C/C (female and male corn) and R/R (female and male rice). Different letter above the bars means that the ratio of fertility were significantly different (*p* < 0.05)

The statistical results showed that the cross direction was an important parameter of the statistical model. Indeed, the interaction between the female strains and male strains was significant (*p* < 0.0001). Among the 30 C/C crosses, 29 were fertile, while 25 of R/R crosses led to viable progeny (over 30 crosses, Fig. 2). Fertility between the two within strain crosses were not found significantly different (*p* value of 0.07). Unlike the within strain crosses, the fertility between the two among strain crosses were significantly different (*p* = 0.012). Indeed, only eight couples over 27 C/R crosses produced a viable progeny against 21 couples over 30 crosses for R/C crosses (Fig. 2). Comparison of the two within strain crosses C/C with C/R and R/R with C/R crosses evidenced the low fertility of among strain crosses (*p* < 0.0001 and *p* = 0.00025, respectively). Same results were observed between C/C and R/C crosses (*p* = 0.003) but no difference was found between R/R and R/C crosses (*p* = 0.219). Except for a longer egg incubation period, we did not notice any aberrant feature in the development of these C/R larvae. Though this asymmetry in fertility has already been described by Pashley (1988), it was not reported by Whitford et al. (1988).

### Segregation pattern of a set of microsatellite markers in F2 generation in crosses between and within corn and rice strains

Obtaining a progeny does not preclude the possibility that some genotypes may be absent from it. We tested this hypothesis by following segregation of a set of markers (see “Materials and methods” section). The corresponding results are shown in supplementary table S1. The markers can be classified into three classes according to the way they segregate. The first four i.e. *Sfrugi2*, *Sfrugi33*, *Sfrugi43* and *Sfrugi76* show a Mendelian segregation for both direction of strain crossing. *Sfrugi6* also shows a Mendelian segregation in R/C progeny but the appearance of a highly frequent mutation for this marker in C/R progeny (1.8 10^−1^/F2 progeny) prevents the estimation of its segregation ratio. Three of the markers (*Sfrugi37, Sfrugi38*, *Sfrugi50*) depart from Mendelian expectations in one direction of the crosses only (in the RC direction for *Sfrugi37* and in the reverse C/R cross for *Sfrugi38* and *Sfrugi50*). Three other markers (*Sfrugi11*, *Sfrugi21*, and *Sfrugi29*) show non-Mendelian segregation in the two reciprocal crosses. The last one *Sfrugi25* could not be genotyped unambiguously, probably due to the existence of a null allele.

Because having 45 % of the markers significantly distorted (*p* value <0.05) was not expected in inter-strain crosses, we decided to test segregation of these distorted markers in intra-strain crosses, in order to check if this transmission ratio distortion (TRD) was inherent of the markers, or could reflect, instead, some inter-strains incompatibilities. We managed to control unambiguously three markers, *Sfrugi43*, *Sfrugi50* and *Sfrugi11* that showed a Mendelian segregation in both C/C and R/R crosses. *Sfrugi37* showed a Mendelian segregation in the R/R cross, but was monomorphic in all C/C crosses. *Sfrugi21* and *Sfrugi*29 were also monomorphic in all crosses genotyped (see “Materials and methods” for details). For these latter markers, despite the fact that we could not assess their segregation pattern in intra-strain crosses, we could at least verify that the microsatellites could be unambiguously genotyped for all individuals, excluding genotyping errors as a potential source of segregation distortion. As a conclusion, for at least two of the markers (*Sfrugi11* and *Sfrugi50*), we can postulate that the TRD is due to inter-strain genetic incompatibilities (Table 1).

**Table 1:** Synthetic table showing segregation patterns (the ones distorted from Mendelian expectation marked with a cross label) for 12 microsatellites markers within F2 progeny from within-strain crosses (C/C: female corn with male corn and R/R: female rice with male rice) and inter-strain crosses in both C/R (female corn with male rice) and R/C (female rice with male corn) cross direction

Marker	F2_INTER		F2_INTRA	
	C/R	R/C	Corn	Rice
*Sfrugi2*	✓	✓		
*Sfrugi33*	✓	✓		
*Sfrugi76*	✓	✓		
*Sfrugi43*	✓	✓	✓	✓
*Sfrugi6*	?	✓		
*Sfrugi37*	✓	✗		✓
*Sfrugi50*	✗	✓	✓	✓
*Sfrugi38*	✗	✓	monomorphic	ambiguous
*Sfrugi11*	✗	✗	✓	✓
*Sfrugi21*	✗	✗	monomorphic	monomorphic
*Sfrugi29*	✗	✗	monomorphic	monomorphic
*Sfrugi25*	?	?	?	?
N total	80	50	94	94

### Screening for endosymbiotic bacteria

Insects are frequently infected by bacterial endosymbionts that manipulate their host reproduction and that can affect sex-ratio or fertility of crosses (Engelstädter and Hurst 2009; Cordaux et al. 2011). Because these bacteria can cause genetic incompatibilities in hybrids, notably through CI, we tested for the presence of such endosymbionts in both strains.

Among the eight couples of primers used for detecting the presence of endosymbiotic bacteria, all produced negative results although all controls were correct (internal controls and positive samples tested in the same time).

### Genomic environment of distorted markers in corn and rice strains genomes

**Fig. 3:**
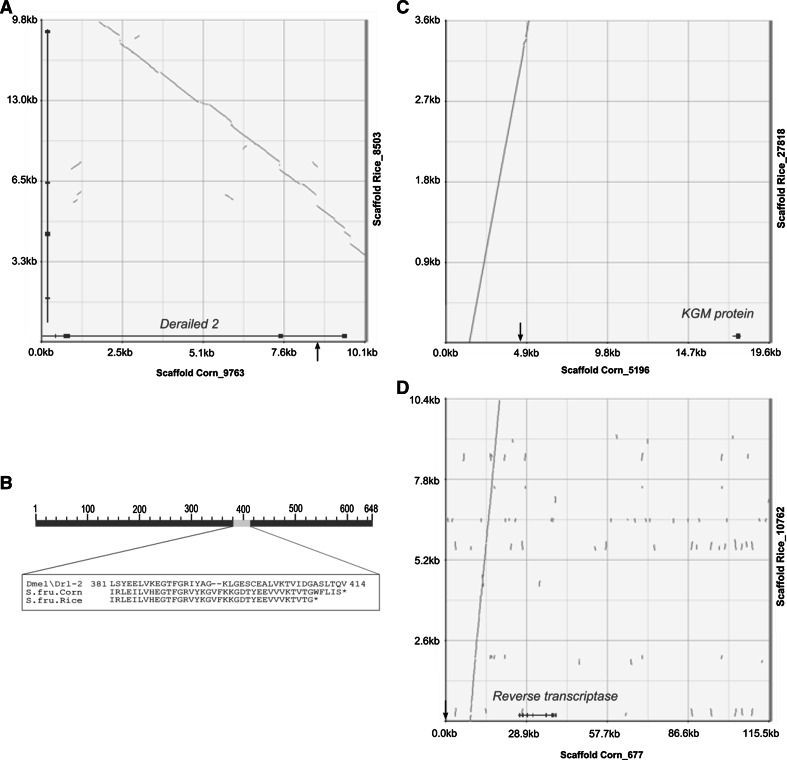
Microsynteny between corn and rice scaffolds around distorted markers. Dotplots resulting from alignments between rice and corn orthologous genomic regions containing distorted microsatellites *Sfrugi11*, *Sfrugi37* and *Sfrugi50* (**a**, **c** and **d** respectively). *Black arrows* indicate the position of the microsatellites. Comparison of the Derailed 2 protein amino acids sequence between *S. frugiperda* corn or rice strain and *D. melanogaster* (**b**)

We investigated the genomic location of the three markers 11, 37 and 50 for which within-strain polymorphic control crosses could be obtained and showed a Mendelian segregation, which led us to conclude that distortion was due to inter-strain incompatibilities. We identified scaffolds matching with these three distorted markers without ambiguity, excluding the fact that these microsatellites are linked to repeated elements (Tay et al. 2010; Fig. 3). *Sfrugi37* is part of a 19.5 kb scaffold in the vicinity of a gene encoding a hypothetical Konjac glucomannan (KGM) protein of *Danaus plexippus*. *Sfrugi50* is carried by a large (115 kb) scaffold devoid of predictions for proteins of known function (except for transposable elements proteins), and *Sfrugi11,* which showed TRD in both crosses direction, is carried by a 10 kb genomic scaffold and overlaps an intervening sequence in the gene encoding a homolog of derailed 2 (*drl2*) of *D. melanogaster*. If this gene is involved in hybrid incompatibility, we expect that the two host-plant strains orthologous proteins should diverge or that the regulatory regions of the gene diverge. When we compared their predicted exonic sequences between corn and rice strain (The FAW Consortium, in preparation), except one mutation generating a premature stop codon (Fig. 3b), we found only synonymous mutations. The scaffold is short (10 kb) and the regulatory region is not available. The rate of synonymous substitution we found between the two orthologs was of 2.8 %. When we compared the closest predicted gene in the vicinity of the non-distorted microsatellite marker *Sfrugi76* between the two strains, we found a rate of 0.5 %. Nucleotidic divergence is thus higher in the vicinity of the distorted marker.

## Discussion

Before investigating the molecular basis of genetic incompatibility between the two *S. frugiperda* strains, we first analyzed criteria reflecting steps in reproductive isolation: fertility of hybrid crosses, F1 hybrid lethality in addition to patterns of meiotic segregation of hybrids in reciprocal second generation (F2) as compared to the meiosis of both parental strains. We found a significant reduction of fertility rate in F1 in C/R cross as compared to R/C, R/R, C/C crosses and a high level of markers showing transmission ratio distortion (TRD) in the F2 progeny obtained by F1 hybrid intercrosses (45 % in C/R cross, 36.6 % in R/C cross). Although the bias in fertility against C/R cross has already been reported by Pashley and Martin (1987), it has also not been confirmed by other studies (Pashley and Martin 1987; Whitford et al. 1988; Quisenberry 1991; Meagher et al. 2004; Groot et al. 2008; Schöfl et al. 2011). These discrepancies may be explained by some heterogeneity in populations that have been used for experimental crosses. Indeed, in our study, the rice strain originates from Florida while the corn strain comes from French Guadeloupe. We cannot neglect the fact that geographic distance may increase, by drift, the genetic distance between the two isolates, however we think that this geographic effect is minor since: (1) *S. frugiperda* is a long-distance migrator which moves annually from the South to the North of the USA; (2) atmospheric trajectories are favorable for the northward transport from the Caribbean to the south-East of the USA and (3) the Florida main haplotypes ratio h4/h2 in the COI gene is conserved in the Caribbean at Puerto-Rico (Nagoshi et al. 2012a). That said, additional phylogeographic studies are required to better assess the level of population structuration between individuals from these localities.

Moreover, a significant level of reproductive isolation between the two stains (Velásquez-Vélez et al. 2011) has been found among individuals within natural populations sampled in central Colombia. Thus, rice and corn strains in this case are very close geographically. Since laboratory populations were used in the present study, one may wonder about the generality of our observations. In their study, Velásquez-Vélez et al. (2011) found that hybrids obtained from individuals recently collected in the wild also exhibited a reduced fitness. Moreover, despite the fact that the laboratory colonies have been reared for several generations, they still display a high level of genetic variation: we have recently shown that 17 out of 21 microsatellite markers are still polymorphic within laboratory populations of either corn or rice strain and show no significant deviation from Hardy–Weinberg expectations in laboratory populations (Arias et al. 2012).

The decrease in fertility rate measured in our study reflects partial embryonic inviability of F1 hybrids obtained in the C/R crosses and may result from partial hybrid incompatibility due to asymmetric parental contribution. For instance maternal inheritance of mitochondria, mRNAs, proteins, and noncoding small RNAs through the maternal cytoplasm may create imbalance in hybrids with the paternally inherited genome. Moreover because the presence of the endosymbiotic bacteria—such as *Wolbachia*—represents a potential cause of cytoplasmic incompatibilities (Kageyama et al. 2012; Brucker and Bordenstein 2012), we investigated the presence of *Wolbachia* and several other bacteria in both corn and rice variants of *S. frugiperda*, but did not detect any of them.

Since F1 hybrids have been obtained in the two reciprocal crosses, we tried to obtain F2 generations through F1 intercross. F1 hybrids were fertile and gave rise to F2 progenies which developed normally showing no obvious phenotypic degeneracy. Nevertheless, since we obtained progeny from the two reciprocal crosses, we have followed the segregation pattern of a set of markers in order to check whether some genotypes would be absent or overrepresented. The high rate of TRD that we found is comparable to the amount of segregation distortion that has been observed within inter-species crosses in other taxa (e.g. *Nasonia* spp., 29 % of markers in adult males (Niehuis et al. 2008); *Arabidopsis lyrata*, 50 % of markers (Kuittinen et al. 2004); *Lepomis* spp., 36.8 % of markers (López-Fernández and Bolnick 2007)). Transmission Ratio Distortion usually occurs at a lower rate in intraspecific than in interspecific crosses (Xianjun et al. 2010) although some exception to the rule has been documented [48 % of distorted markers when crossing highly divergent populations within *Mimulus guttatus* species (Hall and Willis 2005)]. Absence of TRD when crossing the two *S. frugiperda* strains would have argued in favor of absence of F2 degeneracy, while the fact that we found a high level of TRD is consistent with some hybrid incompatibility between the strains at the F2 generation.

The level of TRD is known to increase with genetic distance (Matsubara et al. 2011; Leppälä et al. 2013). Divergence between the two *S. frugiperda* host-plant strains has been estimated to be 2.09 % on average in the COI gene (Kimura 2-parameter distance) by (Kergoat et al. 2012). As a comparison, 1.4 % of base substitution has been found between human and chimpanzee DNA (Britten 2002), between which taxa, divergence raises 4.8 % when including *indels*. Among the genus *Drosophila*, all species pairs separated by a genetic distance of 0.6 % or more (Nei’s (1972) genetic distance D) are completely reproductively isolated (Coyne and Orr 1989). The amount of divergence found between the two strains of *S. frugiperda* is also equivalent to the amount displayed by pairs of differentiated species in the *Spodoptera* genus (Dumas 2013). This divergence, in addition to pre-zygotic barriers to gene flow [reviewed in (Groot et al. 2010)] plus partial F1 hybrid inviability and indirect evidence of F2 hybrid degeneracy through high level of TRD make these two *S. frugiperda* strains more likely “differentiated species” than “host-plant races”.

Within species, TRD can result from competition among male gametes, where sperm with a particular genotype manages to disrupt or outperform their competitors (as in the mouse t-haplotype system and the segregation distorter system in *Drosophila*, (Lyttle 1991; Montchamp-Moreau et al. 2006). In females, the principal opportunity for pre-zygotic distortion occurs during meiosis, when each primary oocyte produces one functional gamete and three polar bodies. This asymmetry provides scope for cheater genotypes to subvert the segregation process in order to improve their chances of appearing in the functional gamete. Finally, after fertilization, embryonic mortality can also lead to transmission distortion even if the rate of loss depends on the genotype. In interspecific crosses, TRD may also result from competition among gametes, due for instance to defects in chromosome segregation during hybrid meiosis (Henikoff et al. 2001; Henikoff and Malik 2002). TRD can also be due to inviability of embryos due to hybrid incompatibilities. Molecular basis involved in hybrid incompatibility can result from Dobzhansky–Muller diverged genes, chromosome rearrangements, sequence divergence, dosage imbalance and/or transposable elements and non-coding repeats, as recently reviewed in (Maheshwari and Barbash 2011).

Dobzhansky–Muller diverged genes model can explain the fact that in *S. frugiperda*, contrary to F2 hybrids, F1 hybrids retain their fitness, if one considers the fact that derived alleles are recessive compared to ancestral alleles (Turelli and Orr 2000). In a two loci model, if the ancestral population *aabb* splits into two sub-populations, one acquiring allele *A* at locus *a*, that becomes fixed *AAbb*, and the others acquiring allele *B* at locus *b*, that becomes fixed *aaBB*. F1 hybrids will be *AaBb*. If *A* is incompatible with *B* but recessive, F1 hybrids will be viable, but some individuals of the F2 progeny will not, due to recombination that renders them homozygous for the two derived incompatible alleles. This model fits well with our observations of high level of TRD in F2 since it provides an explanation for the absence of some genotypes in F2. Since we did not detect sex ratio bias in F1, we suppose that the incompatible loci are carried by autosomes. As opposed to Velázquez-Vélez et al. (2011), in our study sex-ratios are not biased and we do not observe a Haldane’s rule in F1 hybrid progeny.

Transmission ratio distortion loci often cluster in regions of chromosomes that contain hybrid incompatibility genes: an approach for finding incompatibility genes consists in looking for deviation from Mendelian ratio of parental alleles in back cross (BC) or F2 population. This method has been applied widely in seed-bearing plants (Xu et al. 1997; Harushima et al. 2002), and *Nasonia* wasps (Gadau et al. 1999; Niehuis et al. 2008). Therefore, taking advantage of the availability of a first assembly of *S. frugiperda* genome (The FAW Consortium, in preparation), we have mapped distorted microsatellite markers. By synteny with *Bombyx mori,* all these microsatellite markers could be assigned to different autosomal chromosomes. Among the three markers for which distortion could unambiguously be attributed to interstrain incompatibility, only one, *Sfrugi11* is located in the vicinity of a gene of known function encoding homolog of derailed 2 (drl2) of *D. melanogaster*. This gene encodes a shorter peptide in the rice strain as compared to peptide encode in the corn strain. We wondered whether this gene might have contributed to hybrid incompatibility, and looked at its function in early neuronal development. Neurons extend axons over comparatively vast distances to make synaptic connections with their targets. One recently uncovered axon guidance signaling pathway involves interactions between the Wnt (Wingless Integration site) signaling and the Receptor Tyrosine kinase-related tyrosine kinase (Ryk)-like trans-membrane receptor proteins. DRL is a receptor for the Wnt protein, WNT5. DRL binds WNT5 and drl and wnt5 interact during the formation of the embryonic central nervous system (see Fradkin et al. (2010) for review on these receptors). DRL-2 is another receptor which competes with DRL for WNT5 binding at least in the antennal-lobes, but probably not only there since it is expressed in other cell types during early development of *D. melanogaster.* DRL2 and DRL cooperate to establish the olfactory circuitry in *Drosophila* spp. (Sakurai et al. 2009). They form homodimers and can also heterodimerize; this property may be altered in the hybrids since the rice peptide is shorter. Further work is required to show DRL-2 may be involved in hybrid incompatibility. The ongoing *S. frugiperda* genome project, including genomic comparison of the two host-plant strains should shed more light on these and overall genomic regions and their level of differentiation between the two strains.

## Electronic supplementary material

Below is the link to the electronic supplementary material.

Supplementary material 1 (DOCX 34 kb)

Supplementary material 2 (DOCX 23 kb)
