# Supplementary material for: Spodoptera frugiperda (Lepidoptera: Noctuidae) host-plant variants: two host strains or two distinct species?
Source: Genetica. 2015 Feb 19;143(3):305–16. doi: 10.1007/s10709-015-9829-2 (PMC4419160; doi:10.1007/s10709-015-9829-2)
Supplement: Supplementary file 1 — Supplementary material 1 (DOCX 34 kb) [file 10709_2015_9829_MOESM1_ESM.docx]

| Sfrugi2 |  |  |  |  |  |  |  |  |  |  |  |  |  |
| --- | --- | --- | --- | --- | --- | --- | --- | --- | --- | --- | --- | --- | --- |
| Cross # | Parents | |  | Progeny | | | | Expected ratio | p-value |  |  |  |  |
|  | Male | Female |  | AA | AB | AC | CC |  |  |  |  |  |  |
| CR | AC | AA |  | 34 | - | 45 | - | 1:1 | 0,216 |  |  |  |  |
| RC | NA | NA |  | NA | NA | NA | NA | NA | NA |  |  |  |  |
|  |  |  |  |  |  |  |  |  |  |  |  |  |  |
| Sfrugi33 |  |  |  |  |  |  |  |  |  |  |  |  |  |
| Cross # | Parents | |  | Progeny | | | | Expected ratio | p-value |  |  |  |  |
|  | Male | Female |  | AC | AB | BC | BB |  |  |  |  |  |  |
| CR | AB | CB |  | 18 | 20 | 22 | 19 | 1:1:1:1 | 0,931 |  |  |  |  |
| RC | AB | CB |  | 12 | 9 | 10 | 11 | 1:1:1:1 | 0,924 |  |  |  |  |
|  |  |  |  |  |  |  |  |  |  |  |  |  |  |
| Sfrugi76 |  |  |  |  |  |  |  |  |  |  |  |  |  |
| Cross # | Parents | |  | Progeny | | | | | Expected ratio | p-value |  |  |  |
|  | Male | Female |  | AA | AB | AC | BC | CC |  |  |  |  |  |
| CR | AB | AC |  | 21 | 20 | 18 | 21 | - | 1:1:1:1 | 0,960 |  |  |  |
| RC | AC | AC |  | 8 | - | 25 | - | 8 | 1:2:1 | 0,370 |  |  |  |
|  |  |  |  |  |  |  |  |  |  |  |  |  |  |
| Sfrugi43 |  |  |  |  |  |  |  |  |  |  |  |  |  |
| Cross # | Parents | |  | Progeny | | | | | | Expected ratio | p-value |  |  |
|  | Male | Female |  | AA | AB | BB | AC | CC | CB |  |  |  |  |
| CR | AB | AA |  | 36 | 21 | 22 | - | - | - | 2:1:1 | 0,724 |  |  |
| RC | AC | AC |  | 13 | - | - | 22 | 5 | - | 1:2:1 | 0,170 |  |  |
| IntraC | CB | CB |  | - | - | 25 | - | 21 | 45 | 1:1:2 | 0,834 |  |  |
| IntraR | AB | AB |  | 27 | 43 | 24 | - | - | - | 1:2:1 | 0,647 |  |  |
|  |  |  |  |  |  |  |  |  |  |  |  |  |  |
|  |  |  |  |  |  |  |  |  |  |  |  |  |  |
|  |  |  |  |  |  |  |  |  |  |  |  |  |  |
|  |  |  |  |  |  |  |  |  |  |  |  |  |  |
| Sfrugi6 |  |  |  |  |  |  |  |  |  |  |  |  |  |
| Cross # | Parents | |  | Progeny | | | | | | |  | Expected ratio | p-value |
|  | Male | Female |  | AA | AB | BB | AC | DE | DB | EB | EE |  |  |
| CR | AB | AB |  | 8 | 31 | 22 | 14 | - | - | - | - | 1:2:1:0 | ? |
| RC | DE | EB |  | - | - | - | - | 12 | 10 | 14 | 5 | 1:1:1:1 | 0,225 |
|  |  |  |  |  |  |  |  |  |  |  |  |  |  |
| Sfrugi37 |  |  |  |  |  |  |  |  |  |  |  |  |  |
| Cross # | Parents |  |  | Progeny | | Expected ratio | p-value |  |  |  |  |  |  |
|  | Male | Female |  | AB | BB |  |  |  |  |  |  |  |  |
| CR | AB | BB |  | 32 | 47 | 1:1 | 0,091 |  |  |  |  |  |  |
| RC | AB | BB |  | 9 | 33 | 1:1 | 0,0002 |  |  |  |  |  |  |
| IntraC | BB | BB |  | - | 94 | 1 | 1 |  |  |  |  |  |  |
| IntraR | AB | BB |  | 49 | 45 | 1:1 | 0,680 |  |  |  |  |  |  |
|  |  |  |  |  |  |  |  |  |  |  |  |  |  |
| Sfrugi50 |  |  |  |  |  |  |  |  |  |  |  |  |  |
| Cross # | Parents |  |  | Progeny | | | | | | | | Expected ratio | p-value |
|  | Male | Female |  | AC | AB | BC | BB | CC | AA | DE | DF |  |  |
| CR | AB | CB |  | 12 | 4 | 13 | 21 | - | - | - | - | 1:1:1:1 | 0,009 |
| RC | CB | CB |  | - | - | 22 | 12 | 5 | - | - | - | 2:1:1 | 0,207 |
| IntraC | EF | DD |  | - | - | - | - | - | - | 56 | 38 | 1:1 | 0,063 |
| IntraR | CA | CA |  | 35 | - | - | - | 19 | 18 | - | - | 2:1:1 | 0,959 |
|  |  |  |  |  |  |  |  |  |  |  |  |  |  |
| Sfrugi38 |  |  |  |  |  |  |  |  |  |  |  |  |  |
| Cross # | Parents | |  | Progeny | | | | | Expected ratio | p-value |  |  |  |
|  | Male | Female |  | AB | AC | BB | BC | CC |  |  |  |  |  |
| CR | AB | BC |  | 4 | 12 | 28 | 30 | - | 1:1:1:1 | 1,11E-05 |  |  |  |
| RC | BC | BC |  | - | - | 8 | 15 | 15 | 1:1:2 | 0,12 |  |  |  |
| IntraC | CC | CC |  | - | - | - | - | 29 | 1 | 1 |  |  |  |
|  |  |  |  |  |  |  |  |  |  |  |  |  |  |
| Sfrugi11 |  |  |  |  |  |  |  |  |  |  |  |  |  |
| Cross # | Parents | |  | Progeny | | | Expected ratio | p-value |  |  |  |  |  |
|  | Male | Female |  | AA | AB | BB |  |  |  |  |  |  |  |
| CR | AB | BB |  | - | 24 | 52 | 1:1 | 0,001 |  |  |  |  |  |
| RC | AB | AB |  | 18 | 15 | 6 | 1:2:1 | 0,009 |  |  |  |  |  |
| IntraC | AB | AB |  | 13 | 49 | 28 | 1:2:1 | 0,058 |  |  |  |  |  |
| IntraR | AB | AA |  | 28 | 35 | - | 1:1 | 0,378 |  |  |  |  |  |
|  |  |  |  |  |  |  |  |  |  |  |  |  |  |
| Sfrugi21 |  |  |  |  |  |  |  |  |  |  |  |  |  |
| Cross # | Parents | |  | Progeny | | | | Expected ratio | p-value |  |  |  |  |
|  | Male | Female |  | AA | AB | BB | CC |  |  |  |  |  |  |
| CR | AB | AB |  | 4 | 5 | 63 | - | 1:2:1 | <2,2e-16 |  |  |  |  |
| RC | AB | AB |  | 4 | 24 | 12 | - | 1:2:1 | 0,091 |  |  |  |  |
| IntraR | CC | CC |  | - | - | - | 94 | 1 | 1 |  |  |  |  |
|  |  |  |  |  |  |  |  |  |  |  |  |  |  |
| Sfrugi29 |  |  |  |  |  |  |  |  |  |  |  |  |  |
| Cross # | Parents | |  | Progeny | | | Expected ratio | p-value |  |  |  |  |  |
|  | Male | Female |  | AA | AB | BB |  |  |  |  |  |  |  |
| CR | AB | AB |  | - | 16 | 57 | 1:2:1 | <2,2e-16 |  |  |  |  |  |
| RC | AB | AB |  | 8 | 12 | 21 | 1:2:1 | 0,0004 |  |  |  |  |  |

CR=original population 4pop2

RC=original population RC
