# Supplementary material for: Spodoptera frugiperda (Lepidoptera: Noctuidae) host-plant variants: two host strains or two distinct species?
Source: Genetica. 2015 Feb 19;143(3):305–16. doi: 10.1007/s10709-015-9829-2 (PMC4419160; doi:10.1007/s10709-015-9829-2)
Supplement: Supplementary file 2 — Supplementary material 2 (DOCX 23 kb) [file 10709_2015_9829_MOESM2_ESM.docx]

| **Target** | **Gene** | **Primers** | **Sequence (5'-3')** | **Reference** | **Fragment size (pb)** | **Hybridization temperature (°C)** | **Positive control** |
| --- | --- | --- | --- | --- | --- | --- | --- |
| *Rickettsia* | 16S | Rb-F | GCTCAGAACGAACGCTATC | Gottlieb *et al*., 2006 | 900 | 58 | *Bemisia tabaci* infected with *Rickettsia* |
|  |  | Rb-R | GAAGGAAAGCATCTCTGC |  |  |  |  |
| *Rickettsia* | gltA | RicS741F | CATCCGGAGCTAATGGTTTTGC | Davis *et al.*, 1998 (Goodacre *et al.*, 2006) | 450 | 54 | *Bemisia tabaci* infected with *Rickettsia* |
|  |  | RICT1197R | CATTTCTTTCCATTGTGCCATC |  |  |  |  |
| *Arsenophonus* | 16S | ArsF | GGGTTGTAAAGTACTTTCAGTCGT | Duron *et al.*, 2008 | 800 | 52 | *Bemisia tabaci* infected with *Arsenophonus* |
|  |  | ArsR2 | GTAGCCCTRCTCGTAAGGGCC |  |  |  |  |
| *Arsenophonus* | 23S | Ars23S-1 | CGTTTGATGAATTCATAGTCAAA | Thao and Baumann, 2004 | 650 | 52 | *Bemisia tabaci* infected with *Arsenophonus* |
|  |  | Ars23S-2 | GGTCCTCCAGTTAGTGTTACCCAAC |  |  |  |  |
| *Wolbachia* | FtsZ | F2 | TTGCAGAGCTTGGACTTGAA | Vavre *et al.*, 1999 | 400 | 55 | *Drosophila melanogaster* infected with *Wolbachia wMel* (clade A) and *Bemisia tabaci* infected with *Wolbachia* (clade B) |
|  |  | R2 | CATATCTCCGCCACCAGTAA |  |  |  |  |
| *Cardinium* | 16S | CLO-f1 | GGAACCTTACCTGGGCTAGAATGTATT | Gotoh *et al.*, 2007 | 466 | 54 | *Bemisia tabaci* infected with *Cardinium* |
|  |  | CLO-r1 | GCCACTGTCTTCAAGCTCTACCAAC |  |  |  |  |
| *Bacteroidetes* | 16S | ChF | TACTGTAAGAATAAGCACGGC | Zchori-Fein and Perlman, 2004 | 900 | 57 | *Bemisia tabaci* infected with *Cardinium* |
|  |  | ChR | GTGGATCACTTAACGCTTTCG |  |  |  |  |
| *Hamiltonella* | 16S | HbF (92F) | TGAGTAAAGTCTGGGAATCTGG | Zchori-Fein *et al*., 2002 | 800 | 58 | *Bemisia tabaci* infected with *Hamiltonella* |
|  |  | HbR | AGTTCAAGACCGCAACCTC |  |  |  |  |
|  |  |  |  |  |  |  |  |
| Insect | its2 | Its2U | TGTGAACTGCAGGACACATG | Campbell *et al*., 1993 | 550 | 55 | *Bemisia tabaci* |
|  |  | its2L | AATGCTTAAATTTAGGGGGTA | Schilthuizen *et al*., 1998 |  |  |  |
| Eucaryota | COI | COI-LCO | GGTCAACAAATCATAAAGATATTGG | Folmer *et al.*, 1994 | 660 | 47 | *Drosophila melanogaster* |
|  |  | COI-HCO | TAAACTTCAGGGTGACCAAAAAATCA |  |  |  |  |

Campbell BC, Steffen-Campbell JD, Werren J (1993) Phylogeny of the *Nasonia* species complex (Hymenoptera: Pteromalidae) inferred from an internal transcribed spacer (ITS2) and 28s rDNA sequences. Insect Molecular Biology, 2 : 225-237.

Davis MJ, Ying Z, Brunner BR, Pantoja A et al (1998) Rickettsial relative associated with Papaya bunchy top disease. Current Microbiology 26 , 80–84.

Duron O, Bouchon D, Boutin S et al (2008) The diversity of reproductive parasites among arthropods: *Wolbachia* do not walk alone. BMC Biology 6: 1–24.

Folmer O, Black M, Hoeh W et al (1994) DNA primers for amplification of mitochondrial cytochrome c oxidase subunit I from diverse metazoan invertebrates. Mol Marine Biol Biotechnol 3: 294-299.

Gotoh T, Noda H & Ito S (2007): *Cardinium* symbionts cause cytoplasmic incompatibility in spider mites. Heredity 98: 13–20.

Gottlieb Y, Ghanim M, Chiel E et al (2006): Identification and localization of a *Rickettsia sp*. in *Bemisia tabaci* (Homoptera: Aleyrodidae). Appl. Environ. Microbiol. 72: 3646–3652

Schilthuizen M, Nordlander G, Stouthamer R et al (1998) Morphological and molecular phylogenetics in the genus *Leptopilina* (Hymenoptera: Cynipoidea : Eucoilidae). Systematic Entomology, 23 : 253-264

Thao MLL & Baumann P (2004) Evidence for multiple acquisition of *Arsenophonus* by whitefly species (Sternorrhyncha: Aleyrodidae). Curr. Microbiol. 48: 140–144.

Vavre, F, Girin C, and Bouletreau M (1999) Phylogenetic status of a fecundity-enhancing Wolbachia that does not induce thelytoky in Trichogramma. Insect Mol. Biol. 8:67–72.

Zchori-Fein E, Brown JK (2002) **Diversity of prokaryotes associated with** Bemisia tabaci **(Gennadius) (Hemiptera: Aleyrodidae).** Ann Entomol Soc Am 2002, **95:**711-718.

Zchori-Fein E & Perlman SJ (2004) Distribution of the bacterial symbiont *Cardinium* in arthropods. Mol. Ecol. 13: 2009–2016.
